# PLCH1 overexpression promotes breast cancer progression and predicts poor prognosis through the ERK1/2-EGR1 axis

**DOI:** 10.3389/fonc.2025.1577114

**Published:** 2025-05-30

**Authors:** Jing Li, Fenge Jiang, Congcong Wang, Ping Sun, Lei Song, Jiannan Liu

**Affiliations:** ^1^ The 2nd Medical College of Binzhou Medical University, Yantai, China; ^2^ Department of Oncology, The Affiliated Yantai Yuhuangding Hospital of Qingdao University, Yantai, China; ^3^ Department of Geriatric Medicine, The Affiliated Yantai Yuhuangding Hospital of Qingdao University, Yantai, China

**Keywords:** breast cancer, PLCH1, apoptosis, cell cycle, ERK1/2

## Abstract

**Background:**

Phospholipase C η1 (PLCH1), a member of the phospholipase C superfamily, has been implicated in the development of multiple cancers. However, its specific role in breast cancer progression, its association with clinicopathological features, and its prognostic significance remain unclear.

**Methods:**

PLCH1 expression was analyzed across multiple tumor types using the TNMplot database, which integrates RNA-seq, microarray, and normalized data from The Cancer Genome Atlas (TCGA), Genotype-Tissue Expression (GTEx), and Gene Expression Omnibus (GEO), encompassing 40,442 tumor and 15,648 normal samples. Differential expression analysis was performed using boxplots and statistical tests to assess significance. DNA methylation and survival analyses were conducted using TCGA data, with Kaplan-Meier curves and Cox regression to evaluate prognostic value. Functional enrichment analyses, including Gene Ontology (GO) and Kyoto Encyclopedia of Genes and Genomes (KEGG) pathway enrichment, were performed on differentially expressed genes using the clusterProfiler package. Mutation analyses were conducted using mutation annotation format (MAF) files, and pathway activities were correlated with PLCH1 expression via single-sample GSEA (ssGSEA). Experimental validation included immunohistochemistry (IHC) on 100 breast invasive ductal carcinoma samples, real-time quantitative PCR (RT-qPCR), and Western blotting. PLCH1 knockdown functional studies assessed cell proliferation and signaling pathways.

**Results:**

PLCH1 was significantly overexpressed in various cancers, including breast cancer, compared to normal tissues. PLCH1 expression was strongly correlated with the expression of estrogen receptor (ER), progesterone receptor (PR), and human epidermal growth factor receptor 2 (HER2) in breast cancer tissues, further linking PLCH1 to poor prognosis and adverse patient outcomes. Functional studies revealed that PLCH1 was highly expressed in breast cancer cell lines, and PLCH1 knockdown significantly inhibited cell proliferation, induced cell cycle arrest, and reduced cyclin-dependent kinase 1 (CDK1) expression in BT-474 cells. Mechanistically, PLCH1 silencing downregulated early growth response 1 (EGR1) expression by suppressing the extracellular signal-regulated kinases 1 and 2 (ERK1/2) signaling pathway, impairing tumor cell proliferation.

**Conclusions:**

PLCH1 was overexpressed in breast cancer and was associated with worse patient outcomes. Its role in promoting cell proliferation via the ERK1/2-EGR1 axis highlighted PLCH1 as a potential therapeutic target for breast cancer. These findings offer new insights into the molecular mechanisms underlying breast cancer progression and suggest promising avenues for targeted therapy development.

## 1 Introduction

Breast cancer is the most commonly diagnosed cancer among women worldwide and remains the leading cause of cancer-related mortality in this population (1–3). It is a highly heterogeneous disease classified into four major molecular subtypes: triple-negative breast cancer (TNBC), human epidermal growth factor receptor 2 (HER2)-positive, Luminal A, and Luminal B, as defined by the 13th St. Gallen International Brease Cancer Conference (4–6). These subtypes differ in biological characteristics, treatment responses, and prognoses, highlighting the complexity of breast cancer management (7–9). HER2-positive breast cancer accounts for approximately 15–20% of all breast cancer cases (10, 11), and is marked by HER2 overexpression, aggressive tumor behavior, shorter disease-free survival (DFS), and poorer prognosis (12–14). Although HER2-targeted therapies like trastuzumab and pertuzumab have improved outcomes (15), resistance remains a major challenge, necessitating new therapeutic targets (16, 17). The Luminal subtypes, defined by estrogen receptor (ER) and/or progesterone receptor (PR) expression, are the most common form of breast cancer (18, 19). Luminal A tumors (ER+/PR+/HER2−) have low proliferation and favorable prognosis. In contrast, Luminal B tumors (ER+/PR- or low PR/potential HER2-positivity) have worse outcomes (20, 21). Hormonal therapies, including selective estrogen receptor modulators (SERMs) and aromatase inhibitors, have significantly improved survival in ER+ patients (22). However, resistance to endocrine therapy remains a major obstacle (23). Given the diverse biological behaviors and therapeutic challenges associated with HER2-positive and ER+/PR+ breast cancers, a deeper understanding of their molecular mechanisms is essential for advancing personalized treatment approaches and improving patient outcomes.

Phospholipase C (PLC) is a family of enzymes essential for intracellular signal transduction, with various isoforms playing various roles in cellular processes. A wide range of extracellular signals, including hormones (e.g., insulin and growth hormone), growth factors (e.g., epidermal growth factor [EGF] and vascular endothelial growth factor [VEGF]), and lipids, can activate PLC (24). PLC enzymes primarily function as cytoplasmic proteins, selectively hydrolyzing the membrane lipid phosphatidylinositol 4,5-bisphosphate [PI (4, 5) P2] to generate two critical second messengers: inositol 1,4,5-trisphosphate [I (1, 4, 5) P3] and diacylglycerol (DAG). These messengers regulate numerous cellular processes. I (1, 4, 5) P3 triggers calcium release from the endoplasmic reticulum, regulating cell motility, apoptosis, and proliferation, while DAG activates protein kinase C (PKC), influencing signaling pathways such as transcription and cytoskeletal reorganization, thus playing a crucial role in various physiological activities within the cell.

The PLC family includes multiple isoforms, such as PLC-β, PLC-γ, PLC-δ, PLC-ϵ, PLC-ζ, and PLC-η, each with unique regulatory mechanisms and biological functions. Among them, several isoforms have been implicated in tumorigenesis, particularly in breast cancer. For example, PLC-β1 is overexpressed in metastatic breast cancer and promotes tumor cell migration (25). Similarly, PLC-β2 enhances breast cancer cell proliferation by regulating the cell cycle (25, 26). PLC-γ1 regulates Rac1 and CDC42 GTPases through I (1, 4, 5) P_3_-induced calcium release, playing a critical role in breast cancer lung metastasis; inhibiting PLC-γ1 significantly reduces its metastatic potential (27). In contrast, PLC-δ isoforms show divergent roles: PLC-δ1 is downregulated in breast cancer due to hypermethylation and inhibits cell migration by modulating cytoskeletal proteins (28), whereas PLC-δ4 promotes cell division and drives tumor progression (29). These findings highlight the diverse roles of the PLC superfamily in breast cancer, with some isoforms acting as oncogenes and others as tumor suppressor genes.

Phospholipase C η1 (PLCH1), a relatively understudied PLC isoform, was first identified by Hwang in 2005 (30). In recent years, it has gained more attention due to its potential role in tumor biology. Specific single nucleotide polymorphisms at the PLCH1 locus has been associated with an increased risk of squamous cell carcinoma in the Chinese non-smoking populations (31), suggesting that PLCH1 may be related to cancer susceptibility. However, the precise role of PLCH1 in breast cancer remains unclear. The extracellular signal-regulated kinase 1/2 (ERK1/2) pathway, a pivotal effector of the RAS-RAF-MEK-ERK cascade, is aberrantly activated in a substantial proportion (~40-60%) of human cancers through context-dependent mechanisms (32). In lung cancer, constitutive ERK1/2 activation predominantly stems from EGFR mutations (e.g., L858R) or ALK/ROS1 rearrangements, which cause CD8+ T cell suppression and anti-PD-1 resistance (33). Comparatively, pancreatic ductal adenocarcinoma might rely on KRASG12D mutations to hyperactivate ERK1/2, which orchestrates metabolic adaptation to enhance glycolysis while concurrently inhibiting apoptosis (34, 35). Notably, while both malignancies exploit ERK1/2 to promote survival, their upstream triggers differ. For example, ERK1/2 hyperactivation in breast cancer arises through multi-layered mechanisms, including receptor tyrosine kinase signaling via HER2 amplification or EGFR activation, ligand-dependent ERα non-genomic signaling (e.g., membrane-associated SRC/EGFR crosstalk), and epigenetic silencing of ERK1/2 phosphatases (DUSP1/DUSP6) (36–39). Recent studies reveal ERK1/2 promotes breast cancer progression by phosphorylating EGR1 (Ser383/Thr387), enhancing its nuclear translocation and transcriptional activity. Activated EGR1 drives tumorigenesis through coordinated upregulation of pro-proliferation (Cyclin D1), invasion (MMP9/ZEB1), and anti-apoptosis (Bcl-xL) genes, while inducing glycolytic enzymes (HK2/LDHA) for metabolic reprogramming (40–42).

Preliminary evidence indicates that PLCH1 may regulate key signaling pathways, such as the extracellular signal-regulated kinases 1 and 2 (ERK1/2) pathway, which are crucial for tumor proliferation and survival. Additionally, PLCH1 may influence cell cycle progression and apoptosis by modulating proteins such as cyclin-dependent kinase 1 (CDK1), Bcl-2, and Bax.This study aims to elucidate the role of PLCH1 in breast cancer, particularly its expression and functional significance in HER2-positive and ER+/PR+ subtypes. Given the established roles of other PLC isoforms in breast cancer progression, understanding the contribution of PLCH1 to tumor biology may provide novel insights into its potential as a therapeutic target, particularly in aggressive subtypes with poor prognoses.

## 2 Materials and methods

### 2.1 Bioinformatics analysis

To investigate the differential expression of PLCH1 across tumor, normal, and metastatic tissues, RNA sequencing data were analyzed using the TNMplot web tool (https://tnmplot.com/analysis/). TNMplot integrates transcriptomic datasets from The Cancer Genome Atlas (TCGA), Genotype-Tissue Expression (GTEx), and Gene Expression Omnibus (GEO), providing a comprehensive platform for comparing gene expression levels across different tissue types (43). PLCH1 expression was analyzed using default parameters, and comparisons were made between tumor, non-tumor, and metastatic tissues to identify expression patterns relevant to breast cancer progression.

To further explore the prognostic significance of PLCH1 and its relationship with clinicopathological features, the bc-GenExMiner v4.8 platform (http://bcgenex.ico.unicancer.fr) was used (44). This platform integrates transcriptomic and clinical data from large publicly available datasets, enabling correlation analyses between PLCH1 expression and factors such as molecular subtypes, hormone receptor status (ER, PR, human epidermal growth factor receptor 2 [HER2]), and survival outcomes. Univariate and multivariate survival analyses were performed using bc-GenExMiner’s integrated Cox regression and Kaplan-Meier survival modules to assess the prognostic impact of PLCH1 expression in breast cancer patients.

Functional enrichment analyses were conducted to explore the biological pathways and molecular mechanisms associated with PLCH1 expression. Differentially expressed genes were identified based on RNA sequencing data, and Gene Ontology (GO) and Kyoto Encyclopedia of Genes and Genomes (KEGG) pathway enrichment analyses were performed using the clusterProfiler package in R. These analyses highlighted potential pathways regulated by PLCH1, such as cell cycle progression and ERK1/2 signaling.

Methylation analyses of PLCH1 were conducted using TCGA methylation data. CpG island methylation profiles were visualized using heatmaps generated with the pheatmap package, and correlations between methylation levels and clinical characteristics were examined to provide insights into the epigenetic regulation of PLCH1.

Genetic alterations were analyzed using mutation annotation format (MAF) files from TCGA datasets. Mutation frequencies of key genes in high and low PLCH1 expression groups were visualized using the maftools package. Tumor mutational burden (TMB) was also correlated with PLCH1 expression to explore its role in the tumor microenvironment.

Pathway activity was assessed using single-sample Gene Set Enrichment Analysis (ssGSEA). PLCH1 expression was correlated with pathway activity scores to identify key pathways associated with breast cancer progression, such as ERK1/2 signaling and cell cycle regulation. Scatterplots with regression lines were generated to visualize the correlations between PLCH1 expression and specific pathway activities.

Finally, drug sensitivity analyses were conducted using public drug response data (e.g., the Genomics of Drug Sensitivity in Cancer [GDSC] and Cancer Cell Line Encyclopedia [CCLE] databases). Breast cancer cell lines were divided into high and low PLCH1 expression groups, and their responses to commonly used chemotherapeutic agents (e.g., cisplatin and cyclophosphamide) were compared using IC50 values. Statistical analyses (e.g., t-tests or Wilcoxon rank-sum tests) were used to identify significant differences in drug sensitivity.

These bioinformatics analyses provided critical insights into the molecular and clinical significance of PLCH1 in breast cancer, offering a comprehensive understanding of its role in disease progression, prognosis, and therapeutic response.

### 2.2 Kaplan-Meier survival analysis

To evaluate the prognostic significance of PLCH1 expression in breast cancer, survival analyses were conducted using the Kaplan-Meier Plotter (http://kmplot.com/analysis/index.php?p=background) (45). This tool integrates gene expression and survival data from multiple publicly available datasets, including the Gene Expression Omnibus (GEO), European Genome-phenome Archive (EGA), and The Cancer Genome Atlas (TCGA), to provide a comprehensive analysis of the relationship between gene expression and clinical outcomes. The Kaplan-Meier Plotter offers a robust platform for generating survival curves, allowing for stratification of patient samples based on gene expression levels.

For this study, the most appropriate probe set for PLCH1 (214745_at) was selected to accurately measure PLCH1 transcript levels. Patient samples were categorized into two groups (high and low expression) using the optimal cutoff value determined by the software’s algorithm, which minimizes the log-rank P-value for survival differences between the groups. Survival outcomes, including overall survival (OS) and DFS, were analyzed for the PLCH1 expression groups. Hazard ratios (HRs) with 95% confidence intervals (CIs) were calculated using the log-rank test to determine statistical significance. This approach enabled a thorough evaluation of PLCH1 expression as a potential prognostic biomarker in breast cancer and provided insights into its relationship with patient outcomes.

### 2.3 Patients and breast cancer specimens

This study reviewed the clinical data of 100 patients diagnosed with invasive ductal carcinoma of the breast, who were treated at Yantai Yuhuangding Hospital between January 2015 and December 2016 (**Table 1**). All patients underwent standard surgical procedures, followed by routine postoperative treatments, including chemotherapy, radiotherapy, or targeted therapies, in accordance with clinical guidelines.

**Table 1:** Correlations of PLCH1 expression with clinicopathological characteristics.

Characteristics	n	PLCH1		p
		Low	High	
Total	100	47	53	
Age(years)				
<50	31	16	15	0.536
≥50	69	31	38	
ER status				
Negative	31	6	25	<0.01
Positive	69	41	28	
PR status				
Negative	36	10	26	<0.01
Positive	64	37	27	
HER2 status				
Negative	59	39	20	<0.01
Positive	41	8	33	
Molecular Subtypes				
Luminal A	40	27	13	<0.01
Luminal B	29	14	15	
HER2+	18	0	18	
TNBC	13	6	7	
KI67				
	19	11	8	0.290
≥15%	81	36	45	
T stage				
T1	61	30	31	
T2	29	13	16	0.931
T3	7	3	4	
T4	3	1	2	
N stage				
N0	58	30	28	
N1	31	10	21	0.178
N2	7	5	2	
N3	4	2	2	
Menopausal status				
Premenopausal	30	17	13	
Postmenopausal	70	30	40	0.205

Inclusion criteria for the patients were as follows: (1) Patients with histologically confirmed diagnosis of invasive ductal carcinoma; (2) Patients with complete clinicopathological data; (3) Patients without any evidence of distant organ metastasis at the time of surgery; (4) Patients who completed standard postoperative treatment.

Exclusion criteria for the patients were as follows: (1) Patients with incomplete clinicopathological or follow-up data; (2) Patients with the absence of surgery or non-compliance with standard postoperative treatment protocols; or (3) Patients with an undetermined survival status during the follow-up period.

Clinical follow-up was conducted for all patients through 2024. DFS was defined as the time from surgery to the first documented recurrence, either as locoregional relapse or distant metastasis. OS was defined as the time from surgery to death, regardless of cause. Patients who remained alive or disease-free at the time of the last follow-up were censored in the analysis. Vital status and recurrence data were collected through hospital records, direct interviews, and telephone follow-ups to ensure data accuracy and completeness. This comprehensive collection of clinicopathological and follow-up data provided a solid foundation for analyzing the prognostic significance of PLCH1 expression in breast cancer.

### 2.4 Immunohistochemistry

Formalin-fixed, paraffin-embedded tissue samples from breast cancer and adjacent paracancerous tissues were used for IHC analysis. Tissue sections were cut into 4 μm-thick slices and mounted on glass slides. The sections were deparaffinized in xylene and rehydrated through a series of graded ethanol solutions. Antigen retrieval was performed by heating the sections in citrate buffer (pH 6.0) at high temperature for 20 minutes, followed by cooling to room temperature. Endogenous peroxidase activity was blocked by incubating the sections with 3% hydrogen peroxide for 10 minutes.

After rinsing three times with phosphate-buffered saline (PBS), the sections were incubated with 3% bovine serum albumin (BSA) at room temperature for one hour to block nonspecific binding. The sections were then incubated overnight at 4°C with a primary antibody specific for PLCH1. The following day, the slides were washed with PBS and incubated with a secondary antibody conjugated to horseradish peroxidase (HRP) for one hour at 37°C. PLCH1 expression was detected using 3,3′-diaminobenzidine (DAB) as a chromogenic substrate, producing a brown precipitate to indicate positive staining. Nuclei were counterstained with hematoxylin for histological context. After staining, the slides were dehydrated, cleared, and mounted for microscopic evaluation.

PLCH1 expression was assessed semi-quantitatively using a scoring system based on the percentage of positively stained cancer cells. Scores were assigned as follows: 0: <1% of cancer cells stained. 1+: 1–5% of cancer cells stained. 2+: 5–10% of cancer cells stained. 3+: >10% of cancer cells stained. Samples with scores of 1+, 2+, or 3+ were classified as PLCH1-positive, while those with a score of 0 were considered PLCH1-negative. This scoring system provided a consistent framework for evaluating PLCH1 expression across samples, facilitating correlations with clinicopathological features and prognostic outcomes.

### 2.5 Cell lines and cell culture

Human breast cancer cell lines, including MDA-MB-231, MDA-MB-468, MCF-7, SKBR-3, and BT474, as well as the normal breast epithelial cell line MCF-10A, were obtained from Procell Life Science & Technology Co., Ltd. (Wuhan, China). These cell lines represent different molecular subtypes of breast cancer: MDA-MB-231 and MDA-MB-468 are TNBC cell lines; MCF-7 is a luminal-type cell line; SKBR-3 is characterized by HER2 overexpression; and BT474 is a triple-positive breast cancer cell line (ER+, PR+, and HER2+). MCF-10A represents normal, non-tumorigenic breast epithelial cells and served as a control in this study.

Cell culture media and conditions were tailored to meet the specific requirements of each cell line. MDA-MB-231, MDA-MB-468, and MCF-7 cells were cultured in Dulbecco’s Modified Eagle Medium (DMEM; Sparkjade, Shandong, China) supplemented with 10% fetal bovine serum (FBS; Sunncell, China) and 1% penicillin-streptomycin. SKBR-3 cells were cultured in McCoy’s 5A medium (Procell, Wuhan, China) with 10% FBS. BT474 and MCF-10A cells were maintained in their respective complete media as recommended by the supplier (Procell, Wuhan, China).

All cell lines were incubated at 37°C in a humidified atmosphere containing 5% CO_2_. Cell cultures were regularly monitored in a sterile environment to ensure optimal growth conditions and prevent contamination. Subculturing was performed when cells reached 70–80% confluency, and all experiments were conducted using cells in the logarithmic growth phase.

### 2.6 Quantitative real-time RT-PCR

Total RNA was extracted from cells using Trizol reagent (Sparkjade, Shandong, China) following the manufacturer’s protocol. The quality and concentration of RNA were assessed with a spectrophotometer (NanoDrop, Thermo Fisher Scientific), and RNA integrity was verified by agarose gel electrophoresis. Reverse transcription was performed to synthesize complementary DNA (cDNA) from 1 µg of total RNA per sample using the SPARKscript II RT Plus Kit (Sparkjade, Shandong, China) according to the manufacturer’s instructions. Quantitative real-time PCR (qRT-PCR) was conducted using the SYBR Green qPCR Mix Kit with ROX dye (Sparkjade, Shandong, China) on an Applied Biosystems 7500 Real-Time PCR system (Thermo Fisher Scientific). Gene-specific primers were designed to amplify PLCH1 and GAPDH, with GAPDH serving as the internal control for normalization. The primer sequences are listed in **Table 2**.

**Table 2:** Sequences of primers used for RT-qPCR detection.

Target Genes	Forward (5’-3’)	Reverse (5’-3’)
*PLCH1*	TGATAAGAATGGTGACGGCTTGC	TCTCATCTGTGTCGGCTTCCTG
*GAPDH*	GCACCGTCAAGGCTGAGAAC	TGGTGAAGACGCCAGTGGA

The qRT-PCR conditions included an initial denaturation at 95°C for 2 minutes, followed by 40 cycles of 95°C for 15 seconds and 60°C for 30 seconds. Melt curve analysis was performed to confirm the specificity of the amplification. Each sample was analyzed in triplicate, and no-template controls were included in each run to rule out contamination. The relative expression of PLCH1 mRNA was calculated using the 2^−ΔΔCt^ method, where ΔCt represents the difference in threshold cycle (Ct) values between PLCH1 and GAPDH. The fold changes in expression were determined by comparing PLCH1 expression in experimental groups to that in control groups.

### 2.7 Western blotting

Cell or tissue lysates were prepared using radioimmunoprecipitation assay (RIPA) buffer supplemented with 1% protease and phosphatase inhibitors (Sparkjade, Shandong, China). Samples were lysed on ice for 30 minutes, followed by centrifugation at 12,000 × g for 15 minutes at 4°C to remove cellular debris. The supernatants containing total protein were collected, and protein concentrations were determined using a BCA protein assay kit (Thermo Fisher Scientific). For electrophoresis, an appropriate amount of loading buffer was added to the protein lysates, and the mixture was heated at 100°C for 10 minutes to denature the proteins. Equal amounts of protein (20-30 µg) were loaded onto 10% SDS-polyacrylamide gels and separated by electrophoresis. Proteins were then transferred to polyvinylidene fluoride (PVDF) membranes (Sparkjade, Shandong, China) at a constant voltage of 100 V for 90 minutes in transfer buffer containing methanol. After transfer, membranes were blocked with 5% skimmed milk diluted in Tris-buffered saline with Tween 20 (TBST) at room temperature for 1 hour to block nonspecific binding sites. The membranes were then incubated overnight at 4°C with primary antibodies diluted in either 5% skimmed milk or BSA in TBST, according to the antibody manufacturer’s recommendations. The following day, membranes were washed three times with TBST (5 minutes each) and incubated with HRP-conjugated secondary antibodies for 1 hour at room temperature. After incubation, the membranes were washed three more times with TBST, and protein bands were visualized using an enhanced chemiluminescence (ECL) detection system (Sparkjade, Shandong, China). The chemiluminescent signal was captured using a gel imaging system, and band intensities were quantified using ImageJ software (NIH, USA). GAPDH was used as a loading control to ensure equal protein loading across samples.

### 2.8 Small interfering RNA transfection

BT-474 cells were cultured to the logarithmic growth phase and then digested with 0.25% trypsin-EDTA solution (Gibco, USA). The cells were resuspended in complete culture medium and seeded into 6-well plates at a density of 5 × 10^5^ cells per well. After approximately 18 hours of incubation at 37°C with 5% CO_2_, the cells reached 60–70% confluency and were ready for transfection. The plates were incubated at 37°C with 5% CO_2_ for approximately 18 hours to allow cell attachment, ensuring a confluency of 60–70% at the time of transfection. Then, transfection was performed using siRNA targeting PLCH1 and a nonspecific control siRNA (negative control), with Lipo8000 transfection reagent (Beyotime Biotech, Hangzhou, China), following the manufacturer’s instructions. Briefly, siRNA (final concentration: 50 nM) and Lipo8000 were diluted separately in serum-free DMEM, then mixed to form transfection complexes. After incubating the mixture at room temperature for 20 minutes, it was added dropwise to the cells. Cells were incubated with the transfection complex for 6 hours, after which the medium was replaced with fresh complete medium to minimize cytotoxicity. Cells were harvested at designated time points for downstream analyses. Total RNA was extracted 36 hours post-transfection for real-time quantitative PCR (RT-qPCR), and cellular proteins were extracted 48 hours post-transfection for Western blotting. Transfection efficiency was evaluated by fluorescence microscopy or by assessing the knockdown efficiency of target genes through RT-qPCR or Western blotting. All experiments were performed in triplicate to ensure reproducibility.

### 2.9 Cell proliferation assay

Cell proliferation was assessed using a 5-ethynyl-2’-deoxyuridine (EdU) incorporation assay kit (KeyGEN BioTECH, China), which labels proliferating cells by detecting DNA synthesis. Breast cancer cells in the logarithmic growth phase were seeded onto 6-well plates and allowed to adhere overnight. Cells were then treated with 10 μM EdU solution and incubated for 8 hours at 37°C in a humidified atmosphere containing 5% CO_2_. Following incubation, cells were fixed with 4% paraformaldehyde for 15 minutes at room temperature and permeabilized with 0.5% Triton X-100 in PBS for 20 minutes to facilitate reagent access. A Click-iT reaction mix containing Fluor555 dye was prepared according to the manufacturer’s instructions and applied to the cells. Samples were incubated with the Click-iT mix for 30 minutes at room temperature in the dark to avoid photobleaching. After the reaction, cells were washed three times with PBS to remove excess dye. To counterstain the nuclei, cells were incubated with Hoechst 33342 for 10 minutes. EdU-positive cells were identified by their red fluorescence, while total nuclei appeared blue under fluorescence microscopy. Fluorescent images were captured using a fluorescence microscope, and five random fields of view were selected for each sample. The percentage of EdU-positive cells was calculated by dividing the number of red fluorescent nuclei by the total number of Hoechst-stained nuclei. Quantification was performed using ImageJ software (NIH, USA) for consistency and accuracy.

### 2.10 Cell counting kit-8 assay

The (CCK-8) assay (KeyGEN BioTECH, China) was used to evaluate the proliferation of transfected BT-474 cells. After successful siRNA transfection, cells were harvested and resuspended in complete medium. A total of 100 µL of the cell suspension, containing approximately 10,000 cells, was seeded into each well of a 96-well plate. Plates were incubated in a humidified cell culture incubator at 37°C with 5% CO_2_. Cell proliferation was assessed at 0, 24, 48, 72, and 96 hours post-seeding. At each time point, 10 µL of CCK-8 reagent was added to each well. Plates were returned to the incubator and incubated for 1 hour to allow the formation of the formazan product. After incubation, the absorbance at 450 nm was measured using a microplate reader (BioTek, USA), with the absorbance values reflecting the number of viable cells in each well. Each experimental condition was performed in triplicate to ensure reproducibility.

### 2.11 Flow cytometry analysis for apoptosis detection

BT-474 cells were seeded into six-well plates and transfected with siRNA as described previously. After 48 hours of culture under standard conditions (37°C, 5% CO_2_), the cells were harvested for apoptosis analysis. Cells were digested with 0.25% trypsin (EDTA-free) to preserve surface markers, followed by centrifugation at 1,000 × g for 5 minutes at 4°C. The supernatant was discarded, and the cell pellet was washed twice with cold PBS to remove residual media and trypsin. The cell pellet was resuspended in 500 µL of Annexin V Binding Buffer (Beyotime Biotech, China) at a concentration of approximately 1 × 10^6^ cells/mL. Next, 5 µL of Annexin V-FITC and 5 µL of Propidium Iodide (PI) were added to the suspension. The mixture was gently vortexed and incubated at room temperature in the dark for 10 minutes to allow proper staining. After incubation, the samples were immediately analyzed using a flow cytometer (BD FACSCanto II, BD Biosciences) within 1 hour to ensure optimal signal stability. Data acquisition and analysis were performed using FlowJo software (Tree Star, USA).

### 2.12 Statistical analysis

All statistical analyses were performed using GraphPad Prism (version 10.0) and SPSS (version 26.0) software. Continuous variables were presented as mean ± SD, and categorical variables were expressed as frequencies or percentages. A P-value of < 0.05 was considered statistically significant. The chi-square (χ²) test was used to evaluate the association between PLCH1 expression and clinicopathological characteristics, such as tumor grade, stage, and receptor status. For comparisons between two groups, an independent two-tailed t-test was conducted for normally distributed data, while Spearman’s rank correlation was used for nonparametric data. One-way analysis of variance (ANOVA) followed by *post hoc* Tukey’s test was employed for comparisons across multiple groups. Correlations between continuous variables were assessed using Pearson or Spearman correlation coefficients, depending on data distribution. Survival analyses, including OS and DFS, were performed using Kaplan-Meier methods, and differences between survival curves were evaluated with the log-rank test. HRs with 95% CIs were calculated using Cox proportional hazards regression models in univariate and multivariate analyses to identify independent prognostic factors. All statistical tests were two-sided, and graphical representations, including bar graphs, scatter plots, and Kaplan-Meier curves, were generated using GraphPad Prism.

## 3 Results

### 3.1 PLCH1 expression is elevated in breast cancer and correlates with poor prognosis

**Figure 1:**
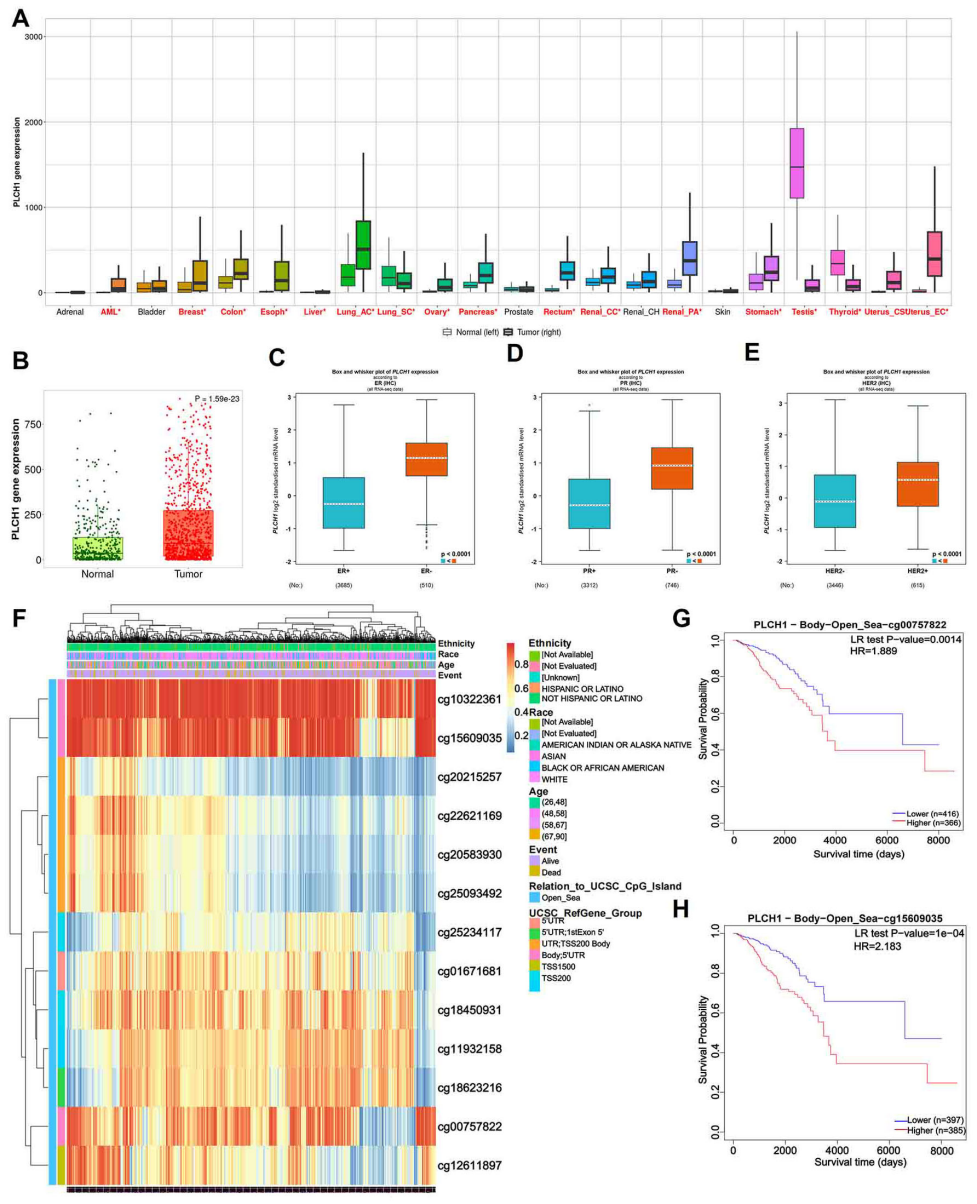
PLCH1 gene expression and its prognostic value in various cancers. **(A)** Boxplot showing PLCH1 expression levels across multiple cancer types compared to corresponding normal tissues. **(B)** Scatter plot of PLCH1 expression in normal (green) and tumor (red) breast tissues. **(C-E)** Boxplots comparing PLCH1 gene expression with key breast cancer-related molecular markers, including ER **(C)**, PR **(D)**, and HER2 **(E)**. Tumor samples positive for these markers (ER+, PR+) exhibited significantly higher PLCH1 expression compared to negative samples (ER−, PR−, P < 0.0001). **(F)** Heatmap of PLCH1 methylation profiles across breast cancer samples. Rows correspond to individual CpG sites, and columns represent patient samples. Annotations include patient ethnicity, age, and survival events. Methylation levels are depicted using a gradient from blue (low methylation) to red (high methylation). **(G, H)** Kaplan-Meier survival curves illustrating the prognostic significance of PLCH1 methylation at specific CpG sites, including cg00757822 (HR = 1.889, P = 0.0014) **(G)** and cg15609035 (HR = 2.183, P = 1e-04) **(H)**. Log-rank test P-values are shown for each comparison. ER, estrogen receptor; BR, progesterone receptor; HER2, human epidermal growth factor receptor 2.

To investigate the role of PLCH1 in breast cancer, its expression pattern across multiple cancer types was analyzed using publicly available datasets. The results showed significantly higher PLCH1 expression in several cancer types, including acute myeloid leukemia (AML), lung cancer, breast cancer, and pancreatic cancer (**Figure 1A**). Notably, PLCH1 was particularly overexpressed in breast cancer compared to normal breast tissues (**Figure 1A**), suggesting a potential oncogenic role in driving tumor initiation and progression. Further analysis confirmed that PLCH1 expression was markedly elevated in breast cancer tissues compared to normal tissues (**Figure 1B**, P = 1.15e-23), emphasizing its importance in breast cancer pathogenesis and its relevance as a target for future investigation.

Stratifying breast cancer samples by clinical and molecular characteristics revealed significant differences in PLCH1 expression. Tumors positive for key breast cancer markers, including ER-positive (ER+) (**Figure 1C**), BRCA protein expression-positive (BR+) (**Figure 1D**), and HER2-negative (HER2-) (**Figure 1E**), exhibited significantly higher PLCH1 expression compared to their respective negative counterparts (ER−, BR−, HER2−) (**Figures 1C-E**, all P < 0.0001). These findings suggest that PLCH1 expression is closely associated with molecular characteristics such as ER, BRCA, and HER2 status, which may contribute to tumor progression. They also underscore its potential as a key molecular marker in breast cancer.

To explore the epigenetic regulation of PLCH1, the methylation patterns across patient subgroups were analyzed. Methylation profiling revealed distinct methylation levels at CpG sites associated with patient ethnicity and clinical outcomes (**Figure 1F**). The heatmap illustrates variations in methylation levels across multiple CpG sites within the promoter and gene body regions of PLCH1, highlighting notable differences among patient subgroups (**Figure 1F**). Specifically, certain CpG sites exhibited higher methylation levels in patients from specific ethnic backgrounds, while others showed differential methylation correlated with clinical events such as disease progression or treatment response (**Figure 1F**). These findings suggest that CpG site-specific methylation of PLCH1 may serve as a key epigenetic regulator of its expression and a potential marker for patient stratification.

Additionally, survival analysis showed that specific CpG site methylation status was strongly linked to patient prognosis. Patients with low methylation at cg00757822 (**Figure 1G**, P = 0.0014) and cg15609035 (**Figure 1H**, P = 1e-04) had significantly worse survival probabilities. This suggests that hypomethylation at these sites could serve as a prognostic biomarker. Collectively, these results highlight the potential of PLCH1 not only as a marker for cancer progression and prognosis but also as a promising therapeutic target due to its association with oncogenic signaling, molecular characteristics, and epigenetic regulation.

### 3.2 High PLCH1 expression correlates with advanced clinical features and poor prognosis in breast cancer

**Figure 2:**
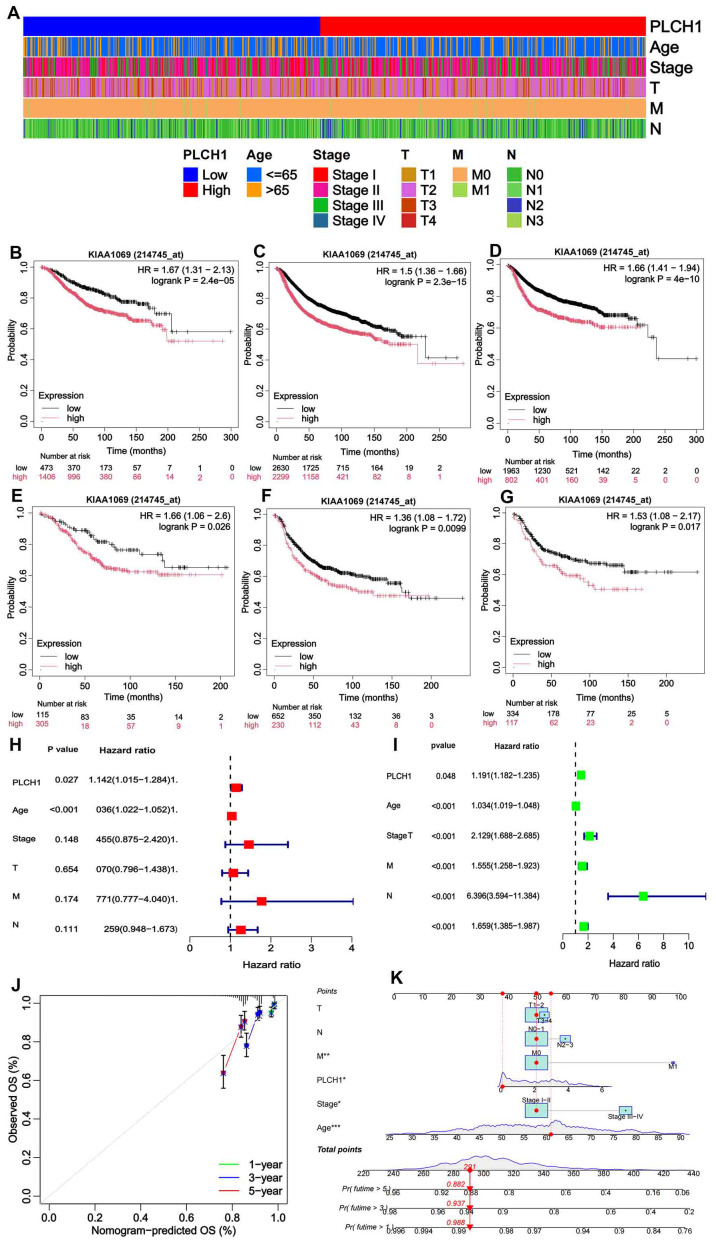
Clinical and prognostic significance of PLCH1 expression in breast cancer. **(A)** Heatmap illustrating patient stratification based on PLCH1 expression (low or high), age (≤65 or >65 years), cancer stage (I-IV), tumor size (T1-T4), metastasis status (M0 or M1), and lymph node involvement (N0-N3). **(B-D)** Kaplan-Meier survival curves comparing OS **(B)**, RFS **(C)**, and DMFS **(D)** between patients with high PLCH1 expression (red line) and low PLCH1 expression (black line). **(E-G)** Kaplan-Meier survival curves comparing OS **(E)**, RFS **(F)**, and DMFS **(G)** between HER2 positive breast cancer patients with high PLCH1 expression (red line) and low PLCH1 expression (black line). **(H)** Univariate Cox regression analysis demonstrates that PLCH1 expression (P = 0.027, HR = 1.142) is a significant risk factor for OS, along with age and cancer stage. **(I)** Multivariate Cox regression analysis shows the combined impact of PLCH1 expression and clinicopathological variables, revealing that advanced stage (Stage III-IV, HR = 2.129), tumor size (T, HR = 1.555), metastasis status (M1, HR = 6.396), and lymph node involvement (N, HR = 1.659) significantly affect patient survival. PLCH1 expression retains statistical significance (P = 0.048) as an independent prognostic factor in the multivariate model. **(J)** Calibration curves for 1-year, 3-year, and 5-year survival predictions based on a prognostic model integrating PLCH1 expression with clinicopathological features. The observed survival rates (dots) closely match the predicted survival rates (lines), indicating the robustness and accuracy of the prognostic model. **(K)** Nomogram for predicting 1-year, 3-year, and 5-year survival probabilities. The nomogram incorporates PLCH1 expression, age, stage, tumor size (T), metastasis status (M), and lymph node involvement (N) into a scoring system. The total score corresponds to a predicted survival probability. High PLCH1 expression and advanced clinical parameters, such as M1 status and Stage III-IV, significantly increase the predicted risk of mortality. OS, overall survival; RFS, relapse-free survival; DMFS, distant metastasis-free survival.

The above analysis indicated the significant role of PLCH1 in the development and progression of breast cancer. To further evaluate its clinical and prognostic significance, its association with clinicopathological characteristics and survival outcomes was examined. Stratification of patients based on PLCH1 expression levels, age, cancer stage, tumor size, metastasis status, and lymph node involvement revealed that high PLCH1 expression was significantly associated with advanced stage, metastasis, and lymph node involvement (**Figure 2A**). These findings suggest that PLCH1 expression is closely linked to key indicators of breast cancer progression, including tumor aggressiveness and metastatic potential.

Kaplan-Meier survival analyses showed that high PLCH1 expression was associated with worse OS (**Figure 2B**), relapse-free survival (RFS) (**Figure 2C**), and distant metastasis-free survival (DMFS) (**Figure 2D**) compared to low PLCH1 expression across all patients (**Figures 2B–D**). Notably, in HER2-positive breast cancer patients, high PLCH1 expression was also correlated with significantly reduced OS (**Figure 2E**), RFS (**Figure 2F**), and DMFS (**Figure 2G**).

To further assess the prognostic role of PLCH1, Cox regression analyses were performed. Univariate Cox regression demonstrated that PLCH1 expression (P = 0.027, HR = 1.142) was a significant risk factor for OS, along with age and cancer stage (**Figure 2H**). Furthermore, multivariate Cox regression analysis confirmed that PLCH1 expression remained an independent prognostic factor (P = 0.048) when adjusted for other clinical variables (**Figure 2I**). Advanced stage (HR = 2.129), tumor size (HR = 1.555), metastasis status (HR = 6.396), and lymph node involvement (HR = 1.659) were also significant contributors to OS (**Figure 2I**).

Given the importance of PLCH1 in breast cancer prognosis, a prognostic model incorporating PLCH1 expression and clinicopathological features was developed and validated. Calibration curves for 1-year, 3-year, and 5-year survival predictions showed excellent concordance between observed and predicted survival rates, confirming the robustness and accuracy of the model (**Figure 2J**). A nomogram was constructed to provide individualized survival predictions by integrating PLCH1 expression, tumor size, metastasis status, lymph node involvement, age, and stage into a scoring system (**Figure 2K**). This tool revealed that high PLCH1 expression, along with advanced stage (Stage III-IV) and metastasis (M1), significantly increased the predicted risk of mortality.

These findings collectively indicate that PLCH1 expression is strongly associated with aggressive clinical features and poor survival outcomes in breast cancer. The integration of PLCH1 expression into prognostic models enhances their predictive accuracy, highlighting its potential as a critical biomarker for risk stratification and individualized treatment planning in breast cancer.

### 3.3 PLCH1 is involved in molecular networks, genomic instability, and drug resistance in breast cancer

**Figure 3:**
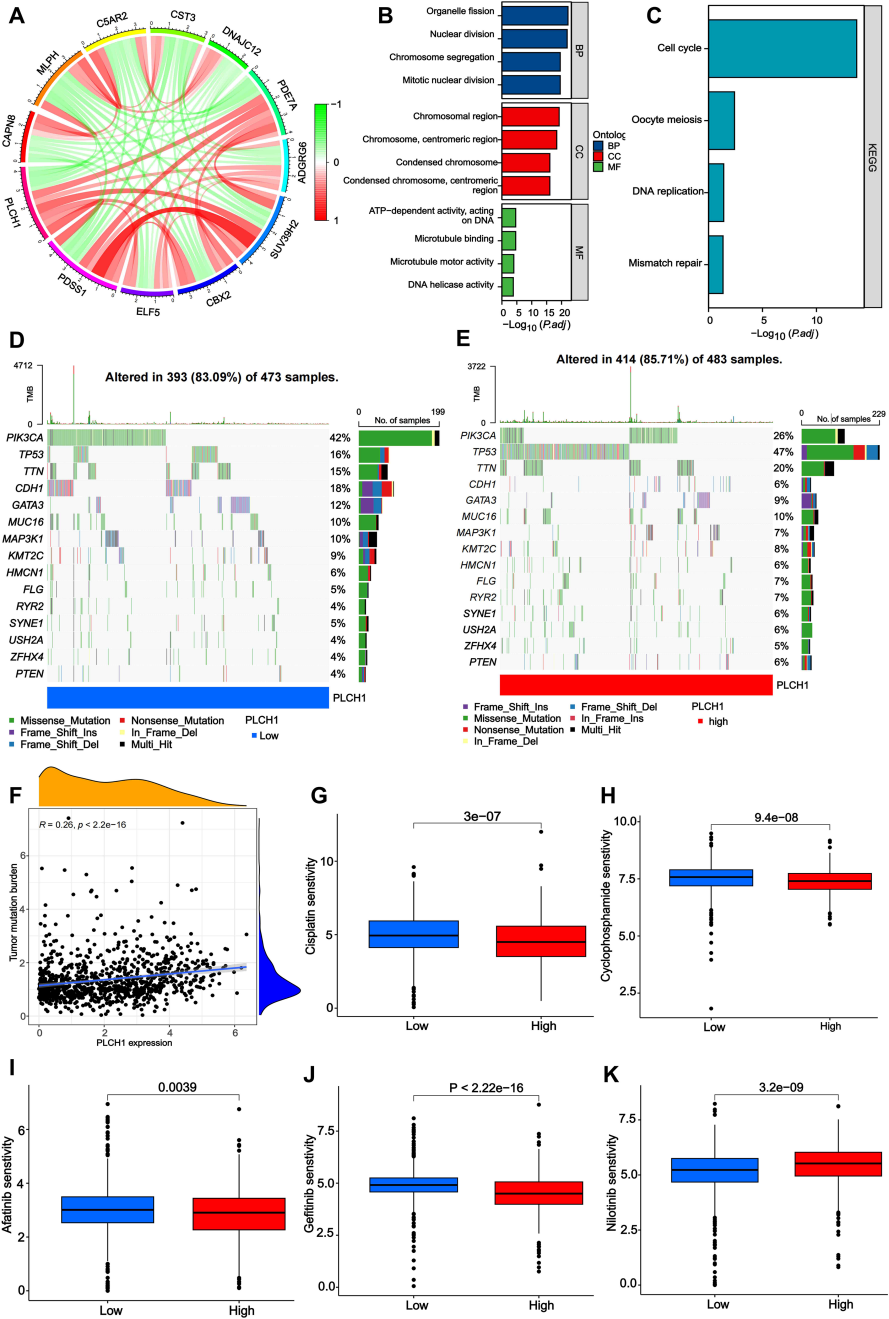
Molecular network, functional enrichment, mutation burden, and drug sensitivity associated with PLCH1 expression in breast cancer. **(A)** Gene interaction network depicting correlations between PLCH1 and other genes, including CAPN6, C5AR2, CST3, and others. Red lines indicate positive correlations, while green lines indicate negative correlations. **(B)** Gene Ontology enrichment analysis of PLCH1-associated genes across three categories: biological processes, cellular components, and molecular functions. **(C)** KEGG pathway enrichment analysis showing that PLCH1-associated genes are significantly enriched in pathways including cell cycle, oocyte meiosis, DNA replication, and mismatch repair. **(D, E)** Oncoprint plots showing mutation frequency of key genes in patient samples with low **(D)** and high **(E)** PLCH1 expression. **(F)** Scatter plot showing a positive correlation between PLCH1 expression and tumor mutation burden (R = 0.26, P < 2.22e-16). This suggests that high PLCH1 expression may be associated with increased genomic instability in tumors. **(G-K)** Drug sensitivity analysis comparing the response of low and high PLCH1 expression groups to various drugs. High PLCH1 expression is associated with reduced sensitivity to Cisplatin (P = 3e-07) **(G)**, Cyclophosphamide (P = 9.4e-08) **(H)**, Afatinib (P = 0.0039) **(I)**, Gefitinib (P < 2.22e-16) **(J)**, and Nilotinib (P = 3.2e-09) **(K)**.

To investigate the functional roles of PLCH1 in breast cancer, a gene interaction network was constructed to identify correlations between PLCH1 and other genes. As shown in **Figure 3A**, PLCH1 is significantly associated with multiple genes, such as CAPN6, C5AR2, and CST3, with positive and negative correlations indicated by red and green lines, respectively (**Figure 3A**). This suggests that PLCH1 participates in a broad molecular network that may influence tumor progression and key signaling pathways.

Next, the GO and KEGG enrichment analyses were performed to explore the biological processes (BPs) and pathways involving PLCH1-associated genes. GO analysis (**Figure 3B**) revealed significant enrichment in BPs such as nuclear division and chromosome segregation, cellular components (CC) like chromosomal and centromeric regions, and molecular functions (MF) including microtubule binding and DNA helicase activity. Similarly, KEGG pathway analysis (**Figure 3C**) indicated that PLCH1-associated genes are enriched in pathways critical to tumor cell proliferation and genomic instability, such as the cell cycle, oocyte meiosis, DNA replication, and mismatch repair. These findings suggest that PLCH1 may promote tumor development by modulating processes essential for cell division, DNA repair, and chromosomal dynamics.

Subsequently, the mutation profiles of key cancer-related genes were analyzed in breast cancer samples with low and high PLCH1 expression levels. In samples with low PLCH1 expression (**Figure 3D**), 83.09% of patients exhibited mutations, with PIK3CA (42%) and TP53 (36%) being the most frequently mutated genes. In samples with high PLCH1 expression (**Figure 3E**), 85.71% of patients exhibited mutations, with an increased frequency of TP53 mutations (47%) compared to low PLCH1 expression samples. These findings suggest that PLCH1 may influence tumor progression through mechanisms involving TP53-related pathways. In addition, a significant positive correlation was observed between PLCH1 expression and TMB (**Figure 3F**, R = 0.26, P < 2.22e-16), indicating that high PLCH1 expression may reflect greater genomic instability in tumors. This relationship underscores the potential role of PLCH1 as a marker of genomic instability in breast cancer.

Finally, the relationship between PLCH1 expression and drug sensitivity was evaluated. High PLCH1 expression was associated with reduced sensitivity to multiple anticancer drugs, including cisplatin (**Figure 3G**, P = 3e-07), cyclophosphamide (**Figure 3H**, P = 9.4e-08), afatinib (**Figure 3I**, P = 0.0039), gefitinib (**Figure 3J**, P < 2.22e-16), and nilotinib (**Figure 3K**, P = 3.2e-09). These results suggest that PLCH1 may contribute to drug resistance in breast cancer, further emphasizing its potential as a predictive biomarker for treatment outcomes. Collectively, these findings reveal that PLCH1 is a key player in molecular networks, genomic instability, and drug resistance in breast cancer. The integration of PLCH1 expression into functional and clinical models may provide valuable insights into tumor biology and aid in the development of personalized therapeutic strategies.

### 3.4 PLCH1 expression correlates with the activation of oncogenic signaling pathways in breast cancer

**Figure 4:**
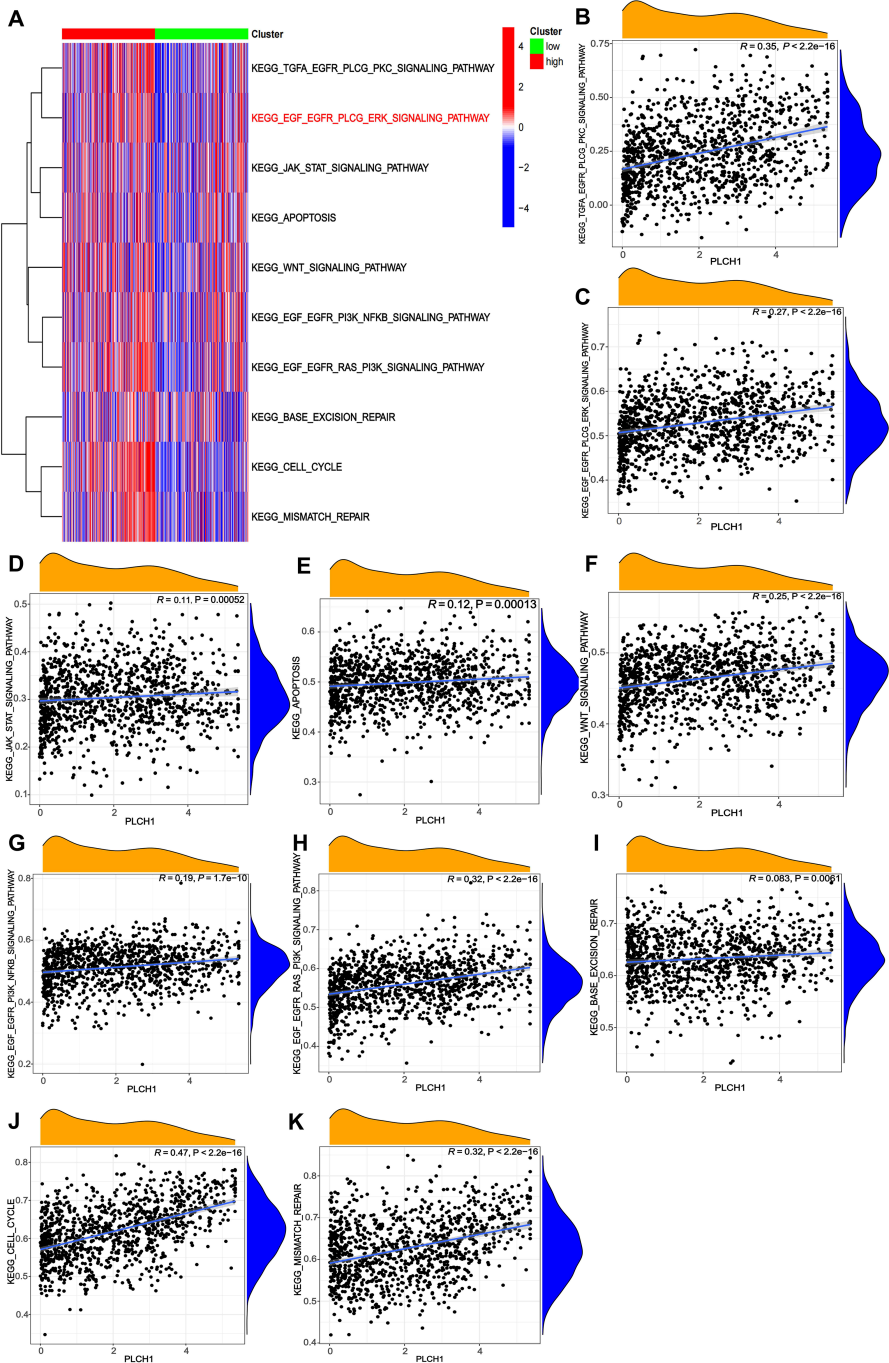
Correlations between PLCH1 expression and KEGG signaling pathway activity in breast cancer. **(A)** Heatmap showing the correlation between PLCH1 expression (high: red; low: green) and the activity of various KEGG signaling pathways, represented by gene set enrichment scores. **(B-K)** Scatter plots illustrating the positive correlations between PLCH1 expression and the activity of specific KEGG signaling pathways, including KEGG_TGFA_EGFR_PLCG_PKC_SIGNALING_PATHWAY (R = 0.35, P < 2.22e-16) **(B)**, KEGG_EGF_EGFR_PLCG_ERK_SIGNALING_PATHWAY (R = 0.27, P < 2.22e-16) **(C)**, KEGG_JAK_STAT_SIGNALING_PATHWAY (R = 0.11, P = 0.00052) **(D)**, KEGG_APOPTOSIS (R = 0.12, P = 0.00013) **(E)**, KEGG_WNT_SIGNALING_PATHWAY (R = 0.25, P < 2.22e-16) **(F)**, KEGG_EGF_EGFR_PI3K_NFKB_SIGNALING_PATHWAY (R = 0.19, P = 1.76e-10) **(G)**, KEGG_EGF_EGFR_RAS_PI3K_SIGNALING_PATHWAY (R = 0.32, P < 2.22e-16) **(H)**, KEGG_BASE_EXCISION_REPAIR (R = 0.083, P = 0.005) **(I)**, KEGG_CELL_CYCLE (R = 0.47, P < 2.22e-16) **(J)**, and KEGG_MISMATCH_REPAIR (R = 0.32, P < 2.22e-16) **(K)**.

Next, the role of PLCH1 in regulating tumor-related signaling pathways was further explored using a pathway enrichment analysis of KEGG signaling pathways. A heatmap (**Figure 4A**) revealed that PLCH1 expression significantly correlates with the activation of multiple pathways. High PLCH1 expression is associated with increased activity in pathways such as KEGG_TGFA_EGFR_PLCG_PKC_SIGNALING_PATHWAY, KEGG_EGF_EGFR_PLCG_ERK_SIGNALING_PATHWAY, KEGG_CELL_CYCLE, and KEGG_MISMATCH_REPAIR. These pathways regulate critical processes in tumor progression, including cell proliferation, apoptosis suppression, and DNA repair, suggesting that PLCH1 may contribute to breast cancer development by modulating these pathways.

To further evaluate the relationship between PLCH1 expression and specific signaling pathways, scatter plots were generated to quantify their correlations. PLCH1 expression showed a strong positive correlation with EGFR-related pathways, including KEGG_TGFA_EGFR_PLCG_PKC_SIGNALING_PATHWAY (**Figure 4B**, R = 0.35, P < 2.22e-16) and KEGG_EGF_EGFR_PLCG_ERK_SIGNALING_PATHWAY (**Figure 4C**, R = 0.27, P < 2.22e-16), which are crucial for tumor cell proliferation and migration. Similarly, PLCH1 expression was positively associated with pathways regulating DNA repair and genomic stability, including KEGG_MISMATCH_REPAIR (**Figure 4K**, R = 0.32, P < 2.22e-16) and KEGG_BASE_EXCISION_REPAIR (**Figure 4I**, R = 0.083, P = 0.005). In addition, PLCH1 expression also correlated strongly with KEGG_EGF_EGFR_RAS_PI3K_SIGNALING_PATHWAY (**Figure 4H**, R = 0.32, P < 2.22e-16) and the KEGG_CELL_CYCLE pathway (**Figure 4J**, R = 0.47, P < 2.22e-16), underscoring its potential role in promoting tumor cell proliferation.

Meanwhile, PLCH1 expression also showed significant associations with other signaling pathways, such as KEGG_WNT_SIGNALING_PATHWAY (**Figure 4F**, R = 0.25, P < 2.22e-16), KEGG_JAK_STAT_SIGNALING_PATHWAY (**Figure 4D**, R = 0.11, P = 0.00052), and KEGG_APOPTOSIS (**Figure 4E**, R = 0.12, P = 0.00013), suggesting its involvement in immune modulation, inflammation, and tumor microenvironment regulation.

Taken together, these results indicate that PLCH1 is closely linked to the activation of key oncogenic pathways, including EGFR-MAPK signaling, cell cycle regulation, and DNA repair processes, all of which play vital roles in tumor progression and genomic stability. The strong correlations between PLCH1 expression and the activity of these pathways highlight its potential as a central regulator in breast cancer biology and a promising therapeutic target.

### 3.5 Elevated PLCH1 expression in breast cancer and its association with poor prognosis

**Figure 5:**
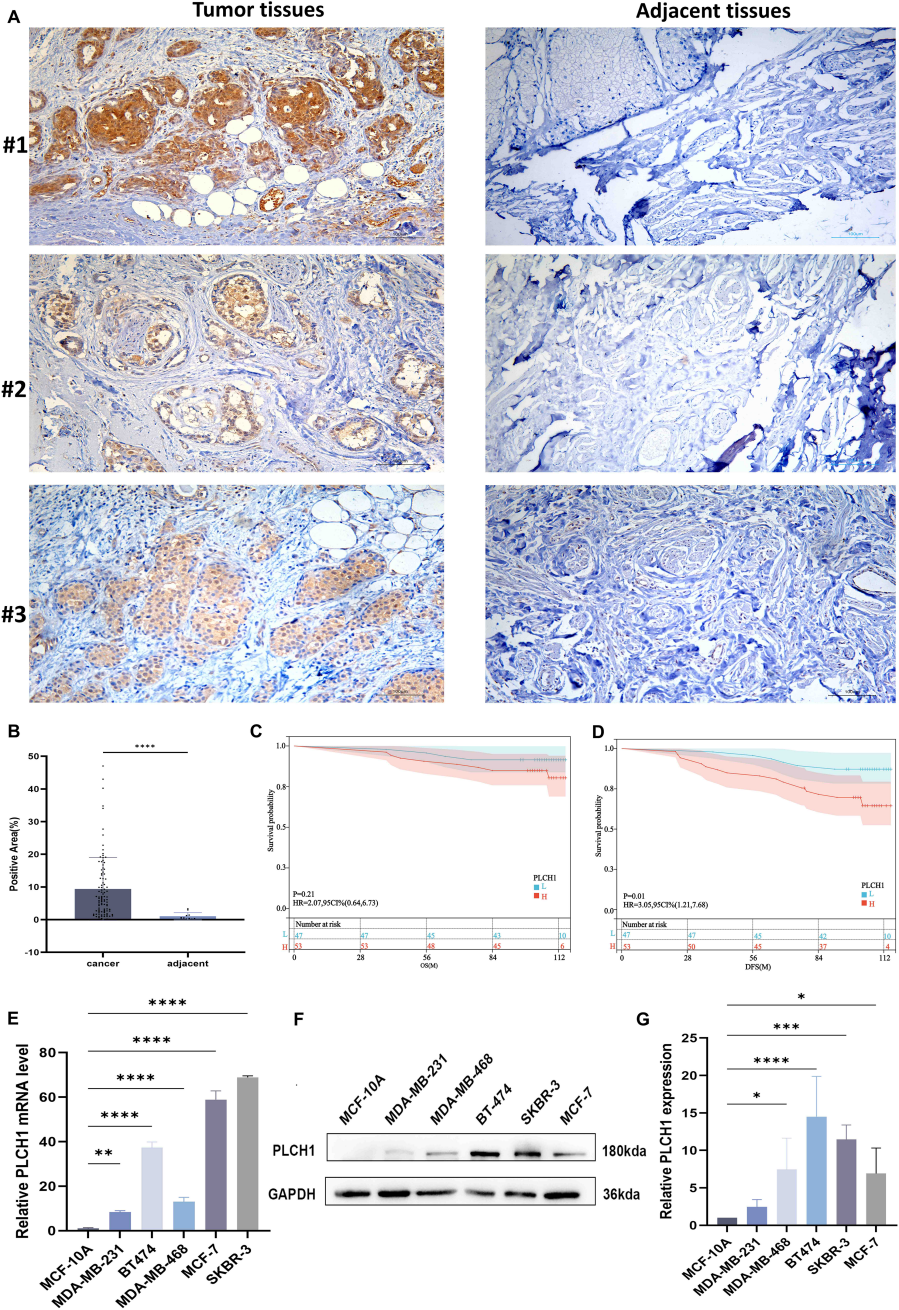
Elevated PLCH1 expression in breast cancer tissues and its association with poor prognosis. **(A)** Representative IHC images of PLCH1 expression in breast cancer tumor tissues (upper panel) and adjacent normal tissues (lower panel) from three patients (#1, #2, and #3). Strong PLCH1 staining (brown) is observed in tumor tissues, while adjacent normal tissues exhibit weak or no staining. **(B)** Quantification of PLCH1-positive staining area in breast cancer tissues and adjacent normal tissues. **(C, D)** Kaplan-Meier survival curves showing the overall survival **(C)** and disease-free survival **(D)** of breast cancer patients with high PLCH1 expression (red line) and low PLCH1 expression (blue line). **(E)** Relative mRNA expression levels of *PLCH1* in normal breast epithelial cells (MCF-10A) and breast cancer cell lines (MDA-MB-231, MDA-MB-468, BT-474, SKBR-3, and MCF-7) analyzed by quantitative PCR (qPCR). **(F)** Western blot analysis of PLCH1 protein expression in MCF-10A and breast cancer cell lines. **(G)** Quantification of relative PLCH1 protein expression normalized to GAPDH. *P < 0.05. **P < 0.01. ***P < 0.001. ****P < 0.0001.

To confirm the findings from our bioinformatics analysis and investigate the clinical significance of PLCH1 in breast cancer, the expression of PLCH1 in tumor and adjacent normal tissues was examined using IHC. Strong PLCH1 staining was observed in tumor tissues, while adjacent normal tissues showed little to no staining (**Figure 5A**). Quantitative analysis confirmed that PLCH1 expression was significantly higher in tumor tissues compared to adjacent normal tissues (**Figure 5B**, P < 0.0001). Next, the prognostic value of PLCH1 expression in breast cancer patients was further assessed using Kaplan-Meier survival analyses. Patients with high PLCH1 expression had worse OS (**Figure 5C**) and DFS (**Figure 5D**) compared to those with low PLCH1 expression, suggesting that PLCH1 is associated with poor prognosis.

To further evaluate the mechanisms underlying PLCH1’s importance in breast cancer, the expression of PLCH1at both the transcriptional and translational levels in breast cancer cell lines using RT-qPCR and Western blotting was examined. Our results revealed significantly upregulated PLCH1 mRNA expression in all breast cancer cell lines (MDA-MB-231, MDA-MB-468, BT-474, SKBR-3, and MCF-7) compared to normal breast epithelial cells MCF-10A (**Figure 5E**, P < 0.01 to P < 0.0001), with the highest expression observed in SKBR-3 and BT-474 cells. Consistent with mRNA expression, Western blot analysis showed elevated PLCH1 protein levels, particularly in SKBR-3 and BT-474 cells (**Figure 5F**). Quantitative analysis confirmed significantly higher PLCH1 protein levels in cancer cell lines compared to MCF-10A (**Figure 5G**, P < 0.05 to P < 0.0001). These findings demonstrate that PLCH1 is highly expressed in breast cancer tissues and cell lines and is strongly associated with poor survival outcomes, highlighting its potential as a prognostic biomarker and therapeutic target in breast cancer.

### 3.6 Knockdown of PLCH1 inhibits breast cancer cell proliferation

**Figure 6:**
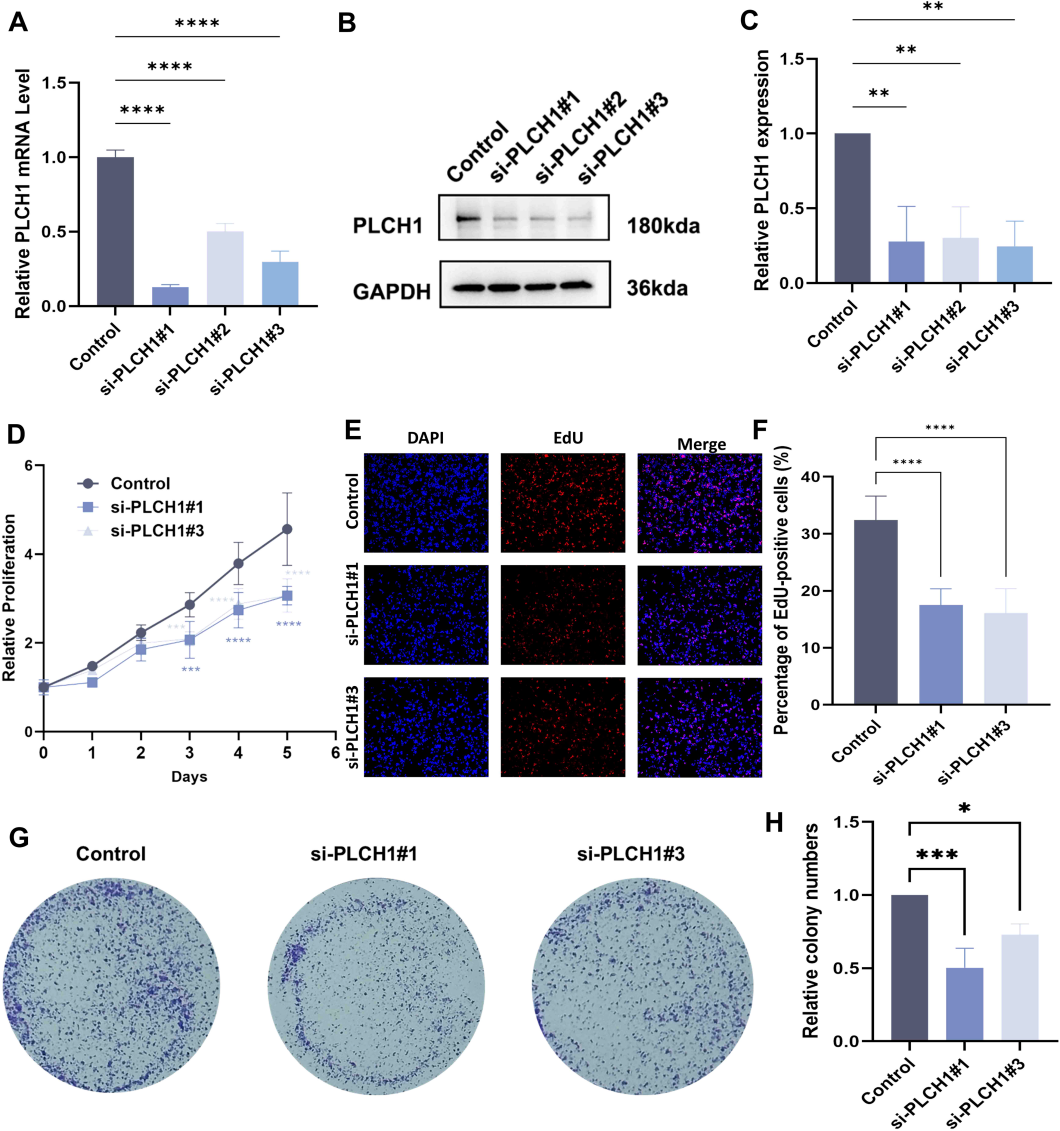
Knockdown of PLCH1 inhibits breast cancer cell proliferation. **(A)** Relative PLCH1 mRNA levels in BT-474 breast cancer cells treated with control siRNA (Control) or three different PLCH1-specific siRNAs (si-PLCH1#1, si-PLCH1#2, and si-PLCH1#3). **(B)** Western blot analysis of PLCH1 protein expression in BT-474 cells treated with control siRNA or PLCH1-specific siRNAs. **(C)** Quantification of PLCH1 protein levels from Western blot results. **(D)** Cell proliferation assay using CCK-8 to evaluate the effect of PLCH1 knockdown on the growth of BT-474 cells over five days. **(E)** EdU incorporation assay to assess DNA synthesis activity in BT-474 cells. Representative images show DAPI-stained nuclei (blue) and EdU-positive cells (red). **(F)** Quantification of EdU-positive BT-474 cells. **(G)** The colony formation assay was used to evaluate clonogenic ability. **(H)** Quantification of the number of cells colony formations. *P < 0.05. **P < 0.01. ***P < 0.001. ****P < 0.0001.

Our bioinformatics analysis suggested that PLCH1 expression is significantly linked to proliferation-related signaling pathways in breast cancer. To evaluate the functional role of PLCH1 in breast cancer cell proliferation, knockdown experiments were performed using three different PLCH1-specific siRNAs in BT-474 breast cancer cells. RT-qPCR analysis revealed that all siRNAs (si-PLCH1#1, si-PLCH1#2, and si-PLCH1#3) significantly reduced PLCH1 mRNA expression compared to the control group (**Figure 6A**, P < 0.0001). Consistent with this, Western blot analysis demonstrated a significant reduction in PLCH1 protein expression in all siRNA-treated groups (**Figures 6B, C**).

To assess the effect of PLCH1 knockdown on cell proliferation a CCK-8 assay was conducted. Cells treated with si-PLCH1#1 and si-PLCH1#3 exhibited significantly reduced proliferation compared to the control group on days 3, 4, and 5 (**Figure 6D**, P < 0.001, P < 0.0001), indicating that PLCH1 is essential for sustaining breast cancer cell growth. Additionally, the DNA synthesis activity of differentially treated cells was evaluated using an EdU incorporation assay. Fewer EdU-positive cells were observed in the si-PLCH1#1 and si-PLCH1#3 groups compared to the control group (**Figure 6E**). Quantitative analysis confirmed that the proportion of EdU-positive cells was significantly reduced in PLCH1-knockdown cells (**Figure 6F**, P < 0.0001). Consistently, the colony formation assay also demonstrated a similar trend (**Figures 6G, H**). These results demonstrate that PLCH1 knockdown effectively suppresses breast cancer cell proliferation and DNA synthesis, underscoring its critical role in tumor cell growth. Collectively, these findings suggest that PLCH1 may serve as a potential therapeutic target in breast cancer.

### 3.7 PLCH1 knockdown induces cell cycle arrest and apoptosis in breast cancer cells

**Figure 7:**
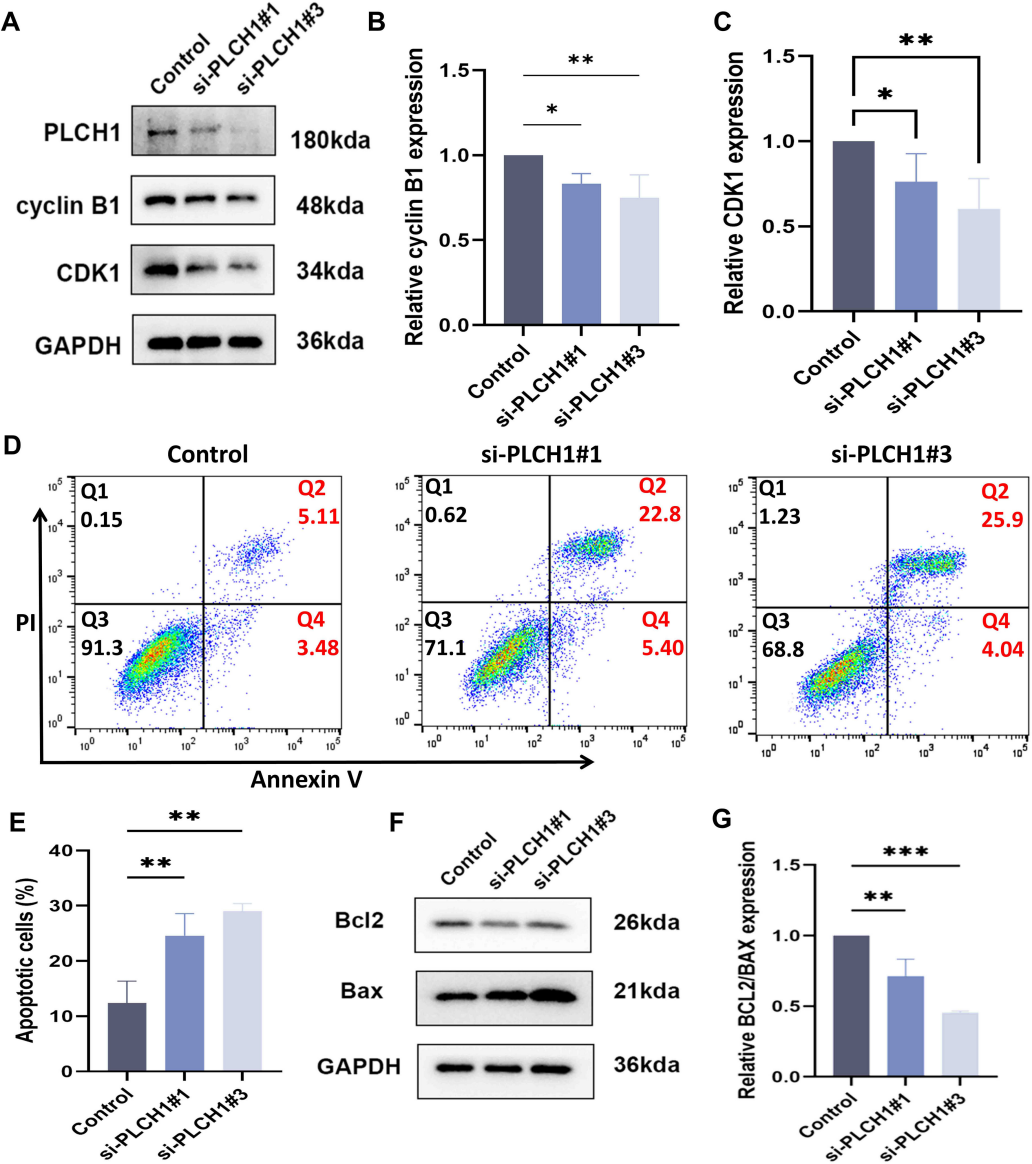
PLCH1 knockdown induces cell cycle arrest and apoptosis in breast cancer cells. **(A)** Western blot analysis of PLCH1, Cyclin B1, and CDK1 protein levels in BT-474 breast cancer cells treated with control siRNA (Control) or PLCH1-specific siRNAs (si-PLCH1#1 and si-PLCH1#3). **(B, C)** Quantification of Cyclin B1 **(B)** and CDK1 **(C)** protein levels based on Western blot results. **(D)** Flow cytometric analysis of cell apoptosis using Annexin V/PI double staining. Q2 (upper right quadrant) represents late apoptotic cells, and Q4 (lower right quadrant) represents early apoptotic cells. **(E)** Quantification of total apoptotic cells (early + late apoptosis) from flow cytometry results. **(F)** Western blot analysis of apoptosis-related proteins Bcl-2 (anti-apoptotic) and Bax (pro-apoptotic). **(G)** Quantification of the Bcl-2/Bax ratio from Western blot results. *P < 0.05. **P < 0.01. ***P < 0.001.

Building on our observation that PLCH1 knockdown inhibits breast cancer cell proliferation (**Figure 6**), its role in regulating the cell cycle and apoptosis in BT-474 cells was further investigated. Knockdown experiments were performed using two specific siRNAs (si-PLCH1#1 and si-PLCH1#3). Western blot analysis showed that PLCH1 knockdown led to a significant reduction in the expression of Cyclin B1 and CDK1, key regulators of cell cycle progression (**Figure 7A**). Quantitative analysis of Western blot results confirmed that Cyclin B1 (**Figure 7B**) and CDK1 (**Figure 7C**) protein levels were significantly decreased in si-PLCH1-treated cells compared to the control group (**Figures 7B, C**, *P < 0.05, P < 0.01), suggesting that PLCH1 knockdown induces cell cycle arrest by downregulating these critical regulators.

Next, the effect of PLCH1 knockdown on apoptosis was evaluated using flow cytometry with Annexin V/PI staining. PLCH1 knockdown significantly increased the proportion of apoptotic cells, with both early (Q4) and late apoptotic cells (Q2) markedly elevated in the si-PLCH1#1 and si-PLCH1#3 groups compared to the control (**Figure 7D**). Quantitative analysis demonstrated a significant increase in total apoptotic cells (early + late apoptosis) in si-PLCH1-treated cells (**Figure 7E**, P < 0.01). To further investigate the molecular mechanisms underlying PLCH1 knockdown-induced apoptosis, the expression levels of Bcl-2 (anti-apoptotic) and Bax (pro-apoptotic) proteins were examined by Western blot. PLCH1 knockdown significantly reduced Bcl-2 levels while increasing Bax levels in BT-474 cells (**Figure 7F**), indicating an activation of the apoptotic pathway. The ratio of Bcl-2/Bax, a critical determinant of apoptosis, was significantly reduced in si-PLCH1-treated cells compared to the control (**Figure 7G**). These findings suggest that PLCH1 promotes breast cancer cell survival and proliferation by regulating the expression of cell cycle-related proteins and suppressing apoptosis. Knockdown of PLCH1 leads to cell cycle arrest and induces apoptosis, highlighting its potential as a therapeutic target in breast cancer.

### 3.8 PLCH1 promotes ERK signaling and regulates apoptosis and cell cycle progression in breast cancer cells

**Figure 8:**
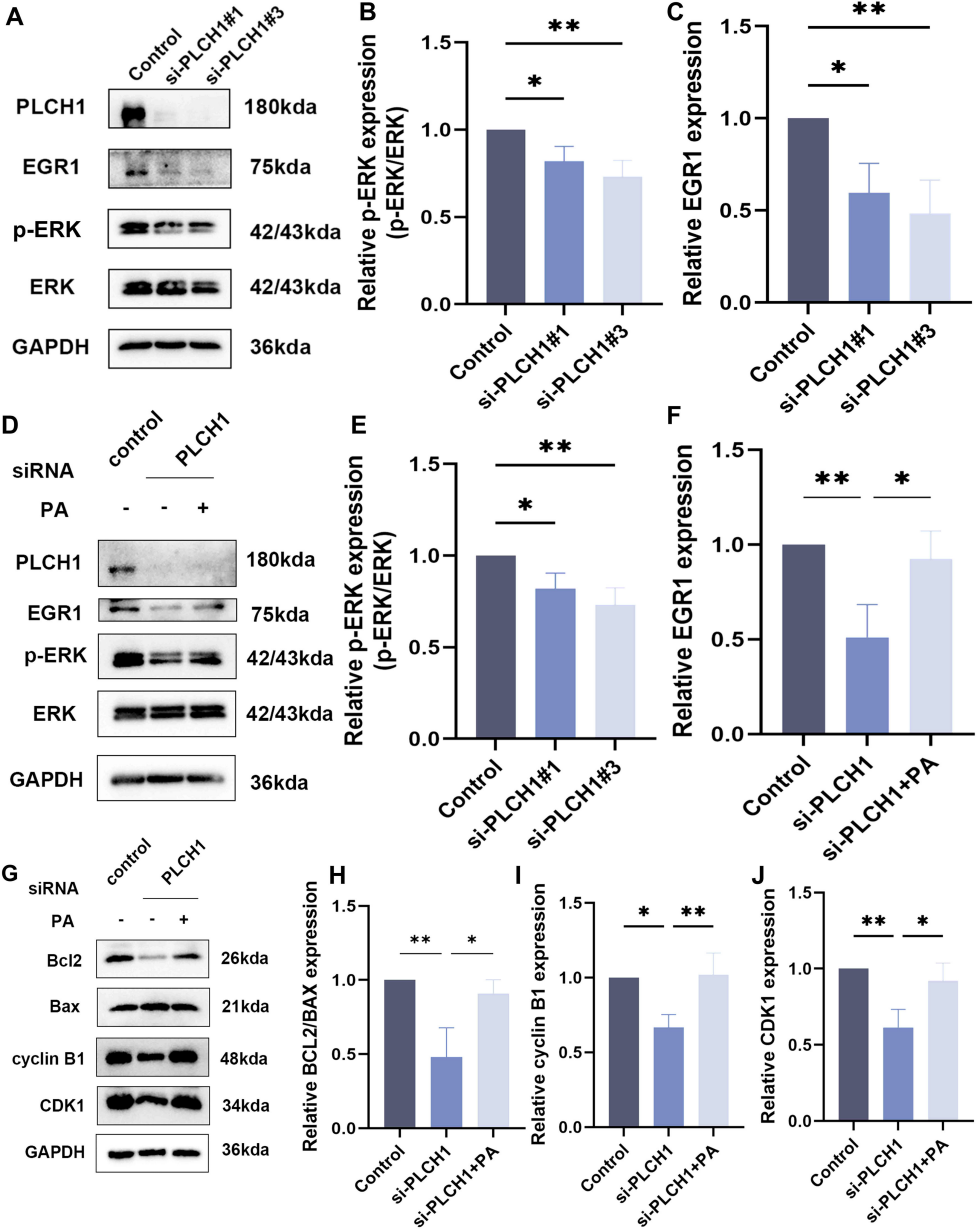
PLCH1 promotes ERK signaling and regulates apoptosis and cell cycle progression in breast cancer cells. **(A)** Western blot analysis of PLCH1, EGR1, and ERK signaling proteins (p-ERK and total ERK) in BT-474 breast cancer cells treated with control siRNA (Control) or PLCH1-specific siRNAs (si-PLCH1#1 and si-PLCH1#3). **(B, C)** Quantification of p-ERK/ERK ratio **(B)** and EGR1 expression **(C)** from Western blot results. **(D)** Western blot analysis of PLCH1, EGR1, and ERK signaling proteins in PLCH1 knockdown cells treated with or without phosphatidic acid. **(E, F)** Quantification of p-ERK/ERK ratio **(E)** and EGR1 expression **(F)** from Western blot results. **(G)** Western blot analysis of apoptosis-related proteins (Bcl-2 and Bax) and cell cycle regulators (Cyclin B1 and CDK1) in PLCH1 knockdown cells treated with or without phosphatidic acid. **(H-J)** Quantification of Bcl-2/Bax ratio **(H)**, Cyclin B1 **(I)**, and CDK1 **(J)** expression from Western blot results. *P < 0.05. **P < 0.01.

To investigate the molecular mechanisms through which PLCH1 regulates breast cancer cell survival and proliferation, its role in the ERK signaling pathway, apoptosis, and cell cycle regulation was analyzed. Knockdown of PLCH1 using two specific siRNAs (si-PLCH1#1 and si-PLCH1#3) in BT-474 breast cancer cells significantly reduced the expression of p-ERK and early growth response 1 (EGR1), key components of the ERK signaling pathway, as demonstrated by Western blot analysis (**Figure 8A**). Quantitative analysis confirmed that the p-ERK/ERK ratio (**Figure 8B**) and EGR1 (**Figure 8C**) protein levels were significantly decreased in PLCH1-knockdown cells compared to the control group (**Figures 8B, C**), suggesting that PLCH1 positively regulates ERK signaling.

To explore whether the inhibition of ERK signaling caused by PLCH1 knockdown could be rescued, PLCH1-knockdown cells were treated with pamoic acid (PA), an activator of the ERK pathway. PA treatment partially restored the expression of p-ERK and EGR1 in PLCH1-knockdown cells (**Figures 8D-F**), indicating a compensatory effect of PA on ERK signaling.

In addition to its effects on ERK signaling, PLCH1 knockdown also influenced apoptosis and cell cycle progression. Western blot analysis revealed that PLCH1 knockdown reduced the expression of the anti-apoptotic protein Bcl-2 while increasing the expression of the pro-apoptotic protein Bax, leading to a decreased Bcl-2/Bax ratio (**Figures 8G, H**). Furthermore, PLCH1 knockdown significantly reduced the expression of Cyclin B1 and CDK1, key regulators of cell cycle progression, suggesting cell cycle arrest (**Figures 8G, I, J**). Importantly, PA treatment partially restored the expression levels of Bcl-2, Cyclin B1, and CDK1 in PLCH1-knockdown cells (**Figures 8G-J**), suggesting that PA can mitigate the effects of PLCH1 knockdown on apoptosis and cell cycle regulation.

In summary, these findings highlight that PLCH1 plays a crucial role in promoting ERK signaling, inhibiting apoptosis, and facilitating cell cycle progression in breast cancer cells. The ability of PA to partially mitigate these effects underscores the potential involvement of ERK signaling in the oncogenic functions of PLCH1 and suggests the existence of a compensatory mechanism that may be targeted for therapeutic intervention.

## 4 Discussion

In this study, a comprehensive analysis of publicly available datasets revealed that PLCH1 was significantly overexpressed in breast cancer tissues compared to normal tissues. IHC staining further confirmed these findings, demonstrating markedly elevated levels of PLCH1 positivity in cancerous tissues from patients with invasive breast carcinoma. The elevated PLCH1 expression was closely associated with poorer clinical outcomes, including reduced OS and DFS, highlighting its potential as a prognostic biomarker. Additionally, an in-depth analysis of clinicopathological features identified significant correlations between PLCH1 expression and key markers of breast cancer, including ER, PR, and HER2. These associations suggest that PLCH1 may play a crucial role in tumor progression, particularly in hormone- and growth factor-driven subtypes.

To further validate these findings, *in vitro* experiments using breast cancer cell lines demonstrated that PLCH1 silencing significantly impaired cell proliferation, suggesting that PLCH1 has a functional role in promoting tumor growth, potentially through its involvement in critical oncogenic pathways. The inhibitory effect of PLCH1 suppression on cancer cell growth further emphasizes its potential as a therapeutic target, particularly for subtypes characterized by high PLCH1 expression.

Growing evidence suggests that members of the PLC family play a critical role in the development and progression of various cancers, including breast cancer. Several PLC isoforms are involved in different oncogenic processes. For instance, PLCϵ-1 promotes lung cancer growth by modulating apoptosis and influences prostate cancer through mitochondrial oxidative metabolism (46, 47). In breast cancer, PLC-β1 (48) and PLC-β2 (49) are abnormally overexpressed, driving tumor progression by enhancing proliferation and migration. Furthermore, PLC-γ1 is associated with poorly and moderately differentiated breast cancers, and its overexpression correlates with increased risk of metastasis and recurrence (50). PLC-γ1 downregulation inhibits breast cancer lung metastasis by blocking Rac1 and CDC42 GTPases via I (1, 4, 5)P_3_-mediated calcium release (51). *In vivo* studies using murine models further confirmed that silencing PLC-γ1 effectively reduced lung metastasis (28). These findings underscore the diverse roles of the PLC family in the biology of breast cancer.

Although the role of the PLC family in breast cancer has been extensively studied, the role of PLCH1 in breast cancer remains largely unexplored. The expression profiles of the PLCH family members show significant differences across various tissues and cell types. Research indicates that PLCH1 is highly expressed in the brain, testis, and ovary, while PLCH2 exhibits higher expression levels in the pancreas and liver. In contrast, PLCH3 has relatively low expression, predominantly detected in certain immune cells (52). Currently, there are numerous studies regarding PLCH1 in the context of tumors, whereas PLCH2 and PLCH3 have not been extensively reported. For instance, in chronic myeloid leukemia, the upregulation of PLCH1 has been confirmed to be associated with cellular resistance to tyrosine kinase inhibitors (53). Furthermore, high expression levels of PLCH1 have been linked to various single nucleotide polymorphisms, which may influence prostate-specific antigen levels, thereby affecting the risk and prognosis of prostate cancer (54). Additionally, studies in ovarian cancer have found that elevated PLCH1 expression can modulate the proliferation and apoptosis of cancer cells (55). This study is the first to demonstrate that PLCH1 promotes breast cancer cell proliferation and contributes to tumor progression, thus filling a significant gap in the research. Our findings establish a significant association between PLCH1 expression and key clinical markers, including ER, PR, and HER2, and further correlate PLCH1 expression with poor patient prognosis. These results suggest PLCH1 as a novel contributor to the pathogenesis of breast cancer and a potential prognostic biomarker.

PLCH1 holds significant potential as both a diagnostic and therapeutic target for breast cancer. Our study demonstrated that elevated PLCH1 expression was closely associated with reduced OS and DFS in postoperative patients with invasive breast cancer. These associations were established through IHC analysis of tissue sections, followed by correlation with clinicopathological parameters and long-term follow-up data. The IHC findings were consistent with bioinformatics analyses, further reinforcing the prognostic value of PLCH1. The association between high PLCH1 expression and poor patient prognosis underscores its potential as a prognostic biomarker.

In addition to its diagnostic implications, our findings suggest that PLCH1 may serve as a novel therapeutic target. Inhibiting PLCH1 expression could be a viable strategy to suppress breast cancer progression, particularly in aggressive subtypes. Our analysis of clinicopathological characteristics revealed a significant association between PLCH1 expression and receptor status in breast cancer, with elevated PLCH1 levels observed in HER2-positive, ER-positive, and PR-positive breast cancer patients. This pattern was consistent with bioinformatics findings. Cellular studies further validated these results, showing significantly higher PLCH1 expression in HER2-positive BT-474 and SKBR3 cell lines compared to other breast cancer cell lines.

Furthermore, the inhibition of PLCH1 led to downregulation of key cell cycle regulators, CDK1 and cyclin B1. Additionally, differential expression of apoptotic regulators Bcl-2 and Bax resulted in a shift toward apoptosis promotion. These findings suggest that PLCH1 plays a critical role in breast cancer cell survival by accelerating cell cycle progression and modulating apoptotic pathways. Our study also indicates that PLCH1 exerts its effects through the classical ERK signaling pathway by regulating EGR1 expression. Previous research has shown that PLCH1 enhances the GPCR/PLC/Ca²^+^ signaling cascade through calcium (Ca²^+^)-mediated activation, leading to ERK activation and subsequent EGR1 regulation (56). R educing nuclear EGR1 levels could significantly inhibit breast cancer cell growth (57, 58). These findings support the involvement of the PLCH1-ERK-EGR1 axis in driving tumor cell proliferation.

In addition to its role in proliferation, EGR1 is also associated with invasion and metastasis. For example, EGR1 has been shown to suppress β-catenin levels via the PTEN-AKT-GSK3β signaling pathway, thereby modulating tumor invasion and metastasis (59). Moreover, EGR1 promotes angiogenesis in other cancer types, further underscoring its multifaceted role in tumor progression (60). Given these findings, PLCH1 may function upstream of EGR1 to regulate multiple oncogenic processes, including proliferation, invasion, and angiogenesis, through its interactions with key signaling pathways. To fully elucidate the mechanistic role of PLCH1 in breast cancer, further studies are needed. Future *in vitro* and *in vivo* research would be required to confirm the interaction between PLCH1 and the ERK-EGR1 pathway and explore its broader connections with additional molecular networks. These studies will provide deeper insights into PLCH1’s role in the biology of breast cancer and may identify novel therapeutic opportunities targeting this axis.

This study highlights the clinical significance of PLCH1 as both a prognostic biomarker and a therapeutic target in breast cancer. By demonstrating that elevated PLCH1 expression was associated with poorer survival outcomes, particularly in HER2-positive and ER-negative/PR-negative subtypes, our findings suggest that PLCH1 could aid in risk stratification and inform tailored therapeutic approaches. Moreover, PLCH1 inhibition may suppress cell proliferation and promote apoptosis by regulating cell cycle proteins (CDK1 and cyclin B1) and the classical ERK-EGR1 signaling axis, further enhancing its potential as a therapeutic target. These results lay the foundation for the development of novel therapies targeting PLCH1, particularly for aggressive breast cancer subtypes with limited treatment options or resistance to current therapies.

However, this study has several limitations. First, although our findings were validated through bioinformatics analysis, IHC staining, and *in vitro* experiments, the lack of *in vivo* models restricted our ability to comprehensively assess the role of PLCH1 in tumor growth, metastasis, and therapy resistance. Second, the clinicopathological correlations were based on a retrospective cohort, which may introduce bias and limit the generalizability of our findings. Third, while we have demonstrated the involvement of PLCH1 in the ERK-EGR1 signaling pathway, further mechanistic studies are required to elucidate its role in other pathways, such as invasion and angiogenesis, and to identify potential interacting partners. In future, larger prospective clinical cohorts with advanced *in vivo* models will be conducted to validate these findings and explore the therapeutic potential of targeting PLCH1 in breast cancer.

## 5 Conclusions

In summary, this study provides compelling evidence that PLCH1 plays a critical role in breast cancer progression, particularly in HER2-positive, ER- positive, and PR-positive subtypes, where its overexpression is associated with poor clinical outcomes. By integrating bioinformatics analyses, IHC staining of patient tissues, and *in vitro* functional studies, we demonstrated that PLCH1 promoted tumor cell proliferation and survival by regulating cell cycle proteins (CDK1 and Cyclin B1) and the ERK-EGR1 signaling pathway. The identification of PLCH1 as a key player in breast cancer biology not only fills a significant gap in understanding this underexplored gene but also highlights its potential as both a prognostic biomarker and therapeutic target.

These findings are of substantial importance, as they pave the way for future research into the mechanistic roles of PLCH1 in breast cancer, including its contributions to invasion, metastasis, and therapy resistance. Additionally, our results underscore the potential of targeting PLCH1 as a novel therapeutic strategy, particularly for aggressive breast cancer subtypes with limited treatment options. These insights hold broad clinical implications and represent a promising step toward improving the prognosis of patients with breast cancer through precision medicine.

## Data Availability

The original contributions presented in the study are included in the article/**Supplementary Material**. Further inquiries can be directed to the corresponding authors.
