# Supplementary material for: PLCH1 overexpression promotes breast cancer progression and predicts poor prognosis through the ERK1/2-EGR1 axis
Source: Front Oncol. 2025 May 30;15:1577114. doi: 10.3389/fonc.2025.1577114 (PMC12162997; doi:10.3389/fonc.2025.1577114)
Supplement: Supplementary file 1 [file DataSheet1.docx]

**PLCH1 overexpression promotes breast cancer progression and predicts poor prognosis through the ERK1/2-EGR1 axis**

Jing Li^1,#^, Fenge Jiang^2,#^, Congcong Wang^2,#^, Ping Sun^2^, Lei Song^3,*^, Jiannan Liu^2,*^

^1^The 2nd Medical College of Binzhou Medical University, 264003, Yantai, China.

^2^Department of Oncology, The Affiliated Yantai Yuhuangding Hospital of Qingdao University, 264000, Yantai, China.

^3^Department of Geriatric Medicine, The Affiliated Yantai Yuhuangding Hospital of Qingdao University, 264000, Yantai, China.

^#^Equal contribution

*Corresponding authors:

Jiannan Liu

Department of Oncology, The Affiliated Yantai Yuhuangding Hospital of Qingdao University, No. 20 Yuhuangding East Road, 264000, Yantai, China. E-mail: [ljnwcc@163.com](mailto:Liujiannansun2013@163.com).

Lei Song

Department of Geriatric Medicine, The Affiliated Yantai Yuhuangding Hospital of Qingdao University, No. 20 Yuhuangding East Road, 264000, Yantai, China. E-mail: [372042212@qq.com](mailto:Liujiannansun2013@163.com).





**Figure S1. PLCH1 knockdown induces cell cycle arrest in breast cancer cells. (A)**. Flow cytometric analysis of cell cycle distribution in BT-474 breast cancer cells treated with control siRNA (Control) or PLCH1-specific siRNAs (si-PLCH1#1 and si-PLCH1#3). **(B)** Quantification of the cell cycle distribution in the G0/G1, S, and G2/M phases.*, P < 0.05.





**Figure S2. PLCH1 overexpression activates the ERK/EGR1 signaling axis, inhibiting apoptosis and promoting cell cycle progression in breast cancer cells. (A)**. Western blot analysis of PLCH1, Cyclin B1, and CDK1 protein levels in BT-474 breast cancer cells transfected with either the vector (Vector) or the PLCH1 overexpression vector (OE). **(B)** Quantification of PLCH1, EGR1, p-ERK/ERK, Bcl-2/BAX, CDK1, and cyclin B1 protein levels based on Western blot results. *, P < 0.05. **, P < 0.01. ***, P < 0.001.

**

Figure S3. (A)**. Western blot analysis of PLCH1, and E-cadherin protein levels in BT-474 breast cancer cells treated with control siRNA (Control) or PLCH1-specific siRNAs (si-PLCH1#1 and si-PLCH1#3). **(B)** Quantification of PLCH1, and E-cadherin protein levels based on Western blot results. ns, difference is not significant.





**Figure S4. PLCH1 knockdown does not affect the migration and invasion abilities of breast cancer cells. (A and C)**. Wound scratch assay and Transwell invasion assay were employed to assess the effect of PLCH1 knockdown on the migration and invasion capabilities of BT-474 cells. **(B and D)** Quantification of cell scratch closure rate and the number of invading cells. ns, difference is not significant. *, P < 0.05. **, P < 0.01. ***, P < 0.001.





**Figure S5. PLCH1 knockdown enhances the sensitivity of breast cancer cells to cisplatin.** CCK-8 assay was performed to evaluate the impact of PLCH1 knockdown on the sensitivity of BT-474 cells to cisplatin (CDDP). * P < 0.05, ** P < 0.01, **** P < 0.001.
